# ^18^F-FDG-PET/CT-based machine learning model evaluates indeterminate adrenal nodules in patients with extra-adrenal malignancies

**DOI:** 10.1186/s12957-023-03184-6

**Published:** 2023-09-26

**Authors:** Lixiu Cao, Dejiang Zhang, Haoxuan Yang, Wengui Xu, Yongliang Liu

**Affiliations:** 1 Department of ECT, Tangshan People’s Hospital, Tangshan, China; 2 Department of Radiology, Tangshan People’s Hospital, Tangshan, Hebei Province China; 3 Department of Urology, The Second Hospital of Hebei Medical University, Shijiazhuang, Hebei Province China; 4 Department of Molecular Imaging and Nuclear Medicine, Tianjin Medical University Cancer Institute and Hospital, National Clinical Research Center for Cancer, Tianjin Key Laboratory of Cancer Prevention and Therapy, Tianjin’s Clinical Research Center for Cancer, Tianjin, China; 5 Department of Neurosurgery, Tangshan People’s Hospital, Tangshan, Hebei Province China

**Keywords:** Indeterminate adrenal nodules, Adrenal metastases, Adrenal benign nodules, PET/CT imaging, Machine learning

## Abstract

**Background:**

To assess the value of an ^18^F-FDG-positron emission tomography/computed tomography (PET/CT)-based machine learning model for distinguishing between adrenal benign nodules (ABNs) and adrenal metastases (AMs) in patients with indeterminate adrenal nodules and extra-adrenal malignancies.

**Methods:**

A total of 303 patients who underwent ^18^F-FDG-PET/CT with indeterminate adrenal nodules and extra-adrenal malignancies from March 2015 to June 2021 were included in this retrospective study (training dataset (*n* = 182): AMs (*n* = 97), ABNs (*n* = 85); testing dataset (*n* = 121): AMs (*n* = 68), ABNs (*n* = 55)). The clinical and PET/CT imaging features of the two groups were analyzed. The predictive model and simplified scoring system for distinguishing between AMs and ABNs were built based on clinical and PET/CT risk factors using multivariable logistic regression in the training cohort. The performances of the predictive model and simplified scoring system in both the training and testing cohorts were evaluated by the areas under the receiver operating characteristic curves (AUCs) and calibration curves. The comparison of AUCs was evaluated by the DeLong test.

**Results:**

The predictive model included four risk factors: sex, the ratio of the maximum standardized uptake value (SUVmax) of adrenal lesions to the mean liver standardized uptake value, the value on unenhanced CT (CTU), and the clinical stage of extra-adrenal malignancies. The model achieved an AUC of 0.936 with a specificity, sensitivity and accuracy of 0.918, 0.835, and 0.874 in the training dataset, respectively, while it yielded an AUC of 0.931 with a specificity, sensitivity, and accuracy of 1.00, 0.735, and 0.851 in the testing dataset, respectively. The simplified scoring system had comparable diagnostic value to the predictive model in both the training (AUC 0.938, sensitivity: 0.825, specificity 0.953, accuracy 0.885; *P* = 0.5733) and testing (AUC 0.931, sensitivity 0.735, specificity 1.000, accuracy 0.851; *P* = 1.00) datasets.

**Conclusions:**

Our study showed the potential ability of a machine learning model and a simplified scoring system based on clinical and 18F-FDG-PET/CT imaging features to predict AMs in patients with indeterminate adrenal nodules and extra-adrenal malignancies. The simplified scoring system is simple, convenient, and easy to popularize.

## Background

The adrenal gland is the fourth most common metastatic site in patients with extra-adrenal cancer, primarily lung cancer (39%) and breast cancer (35%) [1, 2]. Approximately 30% to 70% of adrenal masses incidentally found in cancer patients are metastases [3]. The most common malignant tumour of the adrenal gland is adrenal metastasis (AM) [3]. However, not all adrenal masses can be assumed to represent metastases, and adrenal benign lesions are also not uncommon in cancer patients. Hammarstedt et al. discovered that up to 74% of patients with extra-adrenal malignancy had adrenal benign lesions [4]. Therefore, accurate differential diagnosis of adrenal lesions in cancer patients during follow-up or staging is very important to guide treatment and predict prognosis.

Accurate differential diagnosis of adrenal masses can mostly be performed in patients with extra-adrenal malignancy based on non-invasive imaging techniques, such as magnetic resonance imaging (MRI) and computed tomography (CT). Adrenal washout CT and the value on unenhanced CT images (CTU) have shown perfect ability to differentiate benign from malignant lesions [5–9]. However, the shortcomings of adrenal washout CT, such as a 15-min delayed scanning, lack of sensitivity and additional radiation hazards [10], should not be ignored. In addition, the CTU and adrenal wash-out CT of some benign lesions (hyper-attenuating lesions: CTU ≥ 10 HU) were similar to those of AMs, leading to a misdiagnosis [11, 12]. MRI, especially chemical-shift MRI, has been proven to be the most sensitive examination, but the signal intensity of benign and malignant lesions overlaps considerably [13, 14]. Meanwhile, previous studies have demonstrated that a long diameter (LD) > 3 cm of an adrenal lesion is highly specific for malignancy [15]. Therefore, when patients have unilateral hyper-attenuating (CTU ≥ 10 HU) nodules (LD ≤ 3 cm) based on CT and MRI imaging techniques, it is a challenge to immediately and accurately identify AMs from adrenal benign nodules (ABNs) without additional examinations in cancer patients, and, then, a biopsy may be needed. Although biopsy remains the gold standard for confirmation of the nature of the masses, it is invasive and difficult to perform and thus frequently leads to complications and study failure [16].

Radiomics, as an advanced image analysis technology, has good differential diagnostic ability for adrenal lesions, especially malignant and benign tumors [17, 18]. However, due to the uncertainty of its reliability and the need for computational expertise, radiomics is not widely used in clinical practice. Therefore, it is necessary to explore a non-invasive and simple imaging method for effectively differentiating AMs from ABNs in cancer patients waiting for treatment.

Previous studies have demonstrated that 18F‐FDG‐PET/CT is extremely predictive and sensitive for differentiating adrenal tumours found in routine MRI or CT examinations in patients with or without a cancer history [19–22]. In addition, PET/CT could evaluate the primary lesions and metastases at the same time, so it may be the first and most cost-effective method to characterize adrenal tumours, especially in cancer patients. However, most of the previous studies only explored the value of individual radiological parameters in the differential diagnosis of adrenal tumors [20–22]. In addition, the clinical stages and types of primary cancers have not been comprehensively analyzed. Most importantly, studies on “indeterminate adrenal lesions” based on PET/CT are still rare. Comprehensive differential diagnostic criteria for “indeterminate adrenal lesions” based on machine learning and ^18^F-FDG-PET/CT is needed. Therefore, the purpose of the present study was to assess the accuracy of the predictive model and simplified scoring system based on ^18^F-FDG-PET/CT for differentiating AMs from ABNs in cancer patients with indeterminate adrenal nodules.

## Methods

### Patients

**Fig. 1:**
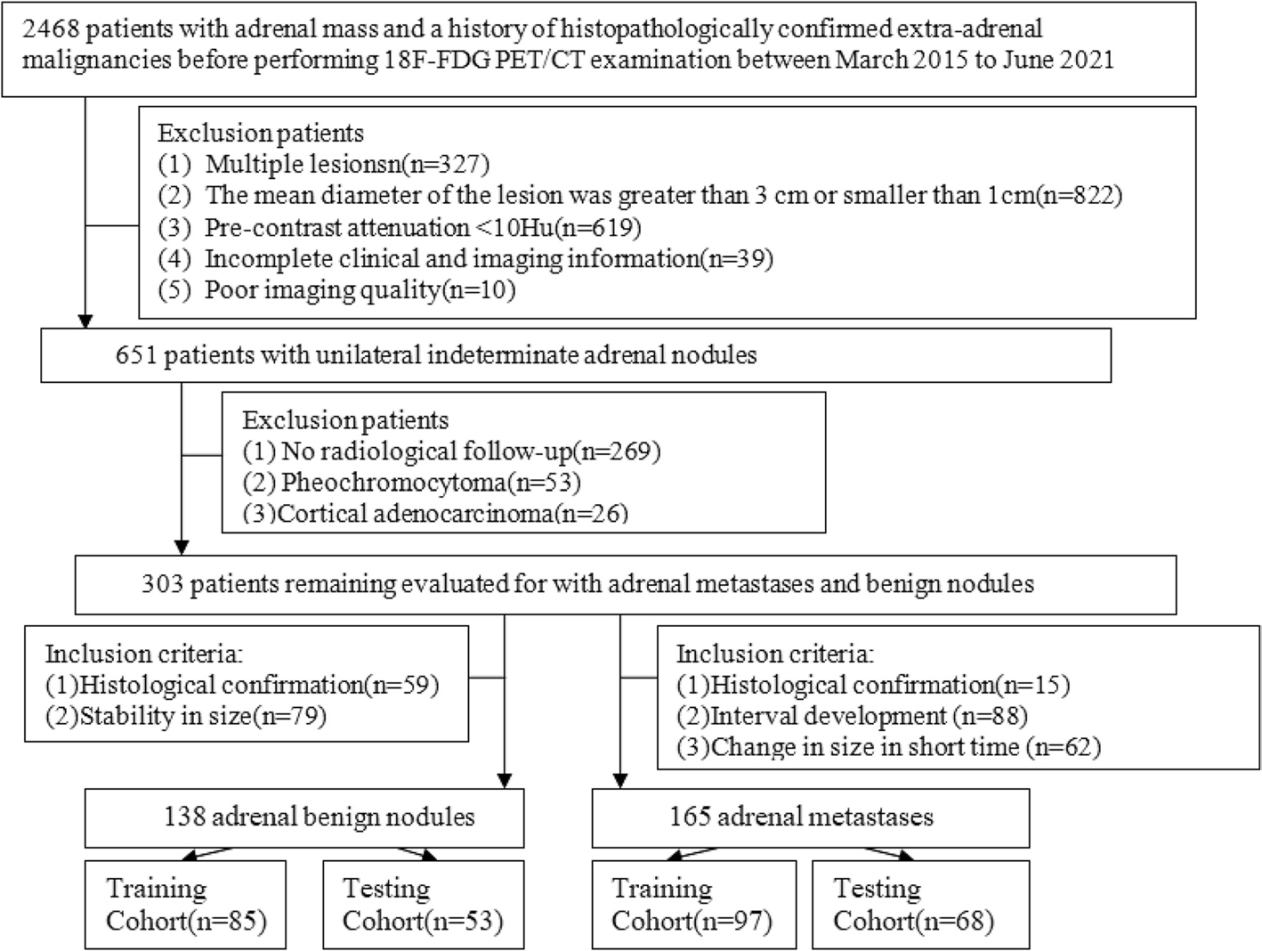
The process of dataset establishment, short time: within 6 months

The Tianjin Medical University Cancer Hospital Institutional Ethics Committee approved our retrospective study. Patients from March 2015 to June 2021 who met the following criteria were enrolled: (1) patients with extra-adrenal malignancy confirmed by histopathology before performing 18F-FDG PET/CT examination; (2) complete PET/CT images and clinical information; and (3) indeterminate adrenal lesions: unilateral hyper-attenuating (CTU ≥ 10 HU) adrenal tumors (1 cm ≤ LD ≤ 3 cm). The reasons for the use of 1 cm as the cut-off for LD were as follows: (a) providing sufficient lesion volume for reliable quantitative measurement technology and (b) increasing confidence in the existence of true focal adrenal lesions. Finally, 165 patients met the eligibility criteria for diagnosing metastases if there is histologic confirmation (*n* = 15), if a new adrenal lesion had developed (*n* = 88) or if the size had increased or decreased after treatment in a short period of time (*n* = 62) [23]. A total of 138 patients met the eligibility criteria for diagnosing benign nodules: histopathological assessment (*n* = 59) or no change in size during at least 1 year of follow-up (*n* = 79). Because the patients in our study comprised a whole dataset, to test the predictive model, we randomly assigned the dataset to a training cohort (*n* = 182; 85 ABNs, 97 AMs) and a testing cohort (*n* = 121; 53 ABNs, 68 AMs) at a ratio of 6:4 (Fig. 1). There was no intersection between the training cohort and testing cohort. Input variables were selected, parameters were adjusted, and the model was fitted on the training cohort. The generalization ability of the model was evaluated on the testing cohort. As the testing cohort is unknown to the model, the accuracy of the evaluation is reliable. Age, sex, and the types and clinical stage of primary cancer were analyzed.

### 18F-FDG PET/CT procedure

The patients fasted for approximately 6 h and had a blood glucose level < 11 mmol/L before undergoing the examination. A Discovery Elite PET/CT scanner (GE healthcare) was used to acquire images approximately 60 min after intravenous injection of ^18^F-FDG (4.2 MBq/kg). Unenhanced CT images were first acquired with 120 kVp, 80 mAs, and a slice thickness of 5 mm from the top of the skull to the middle femur during tidal breathing, and then full-ring dedicated PET images were obtained from the middle thigh to the top of the head during shallow breathing. PET/CT images were reconstructed using the ordered-subset expectation maximization algorithms and CT scans for attenuation correction.

### FDG PET/CT image analysis

**Fig. 2:**
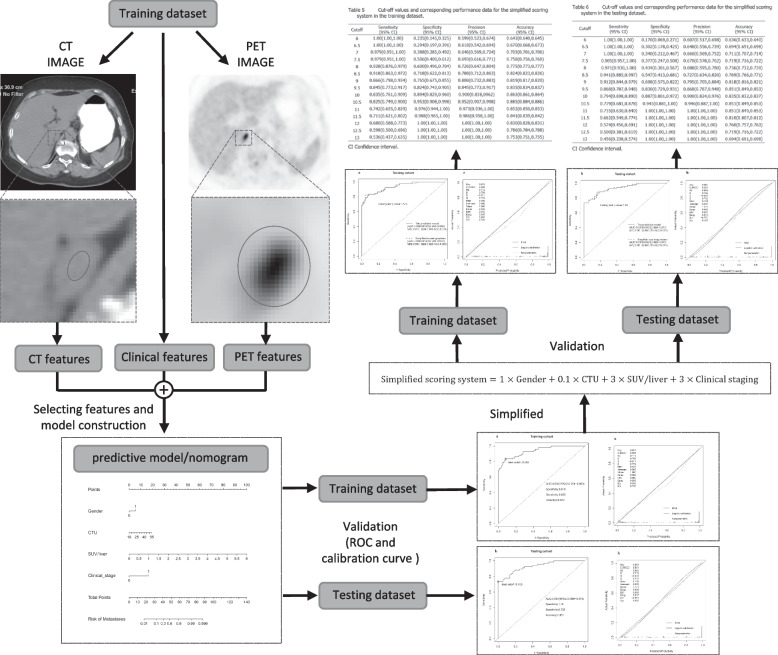
The overall workflow of the development and validation of the predictive model. First, the CT, PET, and clinical features were extracted from the training dataset, and then the predictive model was developed based on significant factors by logistic regression. Second, ROC and calibration curves were used to examine the performance of the nomogram both in the training and testing datasets. Third, a simplified scoring system was built based on the regression coefficients acquired from the training dataset for every individual feature in the predictive model and then the performance of this simplified scoring system was evaluated in both the training and testing datasets. Last, the simplified scoring tables were presented in both the training and testing datasets

A radiologist who had 6 years of PET/CT diagnostic experience and did not know the pathological and clinical information interpreted the CT and PET images. LD, short diameter (SD), left or right and CTU of the adrenal nodules were assessed on the image of the maximum axial area of the tumour. The region of interest (ROI) should contain two-thirds of the largest transverse section of the lesions, and adjacent fat should be avoided when manually measuring CTU. In addition, calcification, hemorrhagic components, and cystic degeneration or necrosis were excluded from the ROI measurements. The maximum standardized uptake value (SUVmax) for each adrenal nodule was recorded by manually drawing a circular oval ROI that included the tumour as much as possible on the axial PET image and while paying attention to avoid adjacent FDG-avid structures. Moreover, the average spleen and liver standardized uptake value (SUV) for each patient was recorded by manually drawing oval ROIs including the spleen or the right lobe of the liver as much as possible on the axial PET images. Then, the ratio of the adrenal lesion SUVmax to the average spleen SUV (SUV/spleen) and the ratio of adrenal lesion SUVmax to the average liver SUV (SUV/liver) were calculated (Fig. 2).

### Predictive modelling and statistical analysis

R (version 4.1.2) was used to perform all statistical analyses. Differences in quantitative characteristics such as SD, LD, age, CTU, SUVmax, SUV/spleen, and SUV/liver were analyzed using the Wilcoxon rank-sum test. For qualitative characteristics such as sex, location, type and clinical stage of primary cancer, the chi-square test was used to analyze whether there was a significant difference between AMs and ABNs. The predictive model was developed based on the above significant factors by logistic regression, which was obtained using the function “lrm” (in the “rms” package). The area under the receiver operating characteristic (ROC) curves (AUCs) of risk factors and the predictive model and simplified scoring system were obtained using the function “reportROC” (in package “reportROC”). The function “nomogram” (in package “rms”) was used to draw the nomograms. The comparison between AUCs was evaluated by the DeLong test in MedCalc. Calibration curves were used to examine the performance characteristics of the nomogram and the simplified scoring system in detail (Fig. 2). *p *values < 0.05 were considered statistically significant.

## Results

### Patient and clinical characteristics

The common extra-adrenal malignancies in the AM group were comprised of lung (72.73%), liver (5.45%), breast (4.24%), and kidney (4.24%) cancers and lymphoma (2.42%) malignancies. The extra-adrenal malignancies in the ABN group included malignancies in the lung (50.0%), gynaecological malignancies (10.87%) and breast cancers (10.14%), thyroid carcinoma (7.24%), and lymphoma (5.07%) (Table 1).

**Table 1:** The types of primary cancer in patients with AMs and ABNs

Primary cancer	AMs** (*n* = 165)** ***n*(%)**	ABNs** (*n* = 138)** ***n*(%)**
Lung cancer	120(72.73%)	69(50%)
liver cancer	9(5.45%)	2(1.45%)
Breast cancer	7(4.24%)	14(10.14%)
Kidney cancer	7(4.24%)	2(1.45%)
Lymphoma	4(2.42%)	7(5.07%)
Gynecological cancer	3(1.82%)	15(10.87%)
Thyroid cancer	1(0.61%)	10(7.24%)
Colorecta cancer	1(0.61%)	3(2.17%)
Gingival cancer	2(1.21%)	2(1.45%)
Esophagus cancer	2(1.21%)	0
Gastric cancer	2(1.21%)	3(2.17%)
Malignant melanoma	2(1.21%)	3(2.17%)
Bladder cancer	0	2(1.45%)
Prostate cancer	0	1(0.73%)
Laryngeal cancer	1(0.61%)	1(0.73%)
Soft tissue sarcoma	3(1.82%)	2(1.45%)
Pancreas cancer	0	1(0.73%)
Tongue cancer	1(0.61%)	1(0.73%)

*AMs* adrenal metastases, *ABNs* adrenal benign nodules

The demographic details of the AM and ABN patients are presented in Table 2. There were no significant differences in any of the variables (sex, SD, LD, age, location, CTU, SUVmax, SUV/liver, SUV/spleen, and the types and clinical stage of primary cancer) (Table 2, *P* > 0.05) between the training and testing datasets, showing the rationality of randomly grouping the total data. Patients with ABNs had a higher female percentage than patients with AMs in both the training (60.0% vs. 28.9%, *p* < 0.001) and testing (62.3% vs. 41.2%, *p* = 0.034) datasets. The AM group had a higher proportion of patients with clinical stage III/IV extra-adrenal cancers, while the ABN group had a lower proportion of those with clinical stage III/IV cancers in both the training (*p* < 0.001) and testing (*p* = 0.007) datasets. For the primary cancer types, the AM group had a higher primary lung cancer: other cancer ratio than the ABN group in both the training (2.88 vs. 0.848, *p* < 0.001) and testing (3.25 vs. 1.208, *p* = 0.012) datasets.

**Table 2:** Clinical and PET/CT characteristics of the patients in the training and testing cohorts

Characteristics	Training cohort (*n* = 182)				Testing cohort (*n* = 121)				*P*^+^value
	Total (*n* = 182)	ABNs (*n* = 85)	AMs (*n* = 97)	*P* value	Total (*n* = 121)	ABNs (*n* = 53)	AMs (*n* = 68)	*P* value	
Age (years)	63[56,68]	64[58;69]	61[56;68]	0.145	62[56;68]	64[58;69]	61[56;67]	0.066	0.954
CTU	32[24;38]	25[19;32]	37[31;40]	< 0.001^*^	33[25;38]	25[16;33]	35 [31;41]	< 0.001^*^	0.879
SUVmax	3.7[2.7;6.9]	2.9[2.2;3.5]	6.6[3.9;9.0]	< 0.001^*^	4.2[2.8;7.0]	2.8[2.4;3.5]	6.5[4.5;9.5.0]	< 0.001^*^	0.417
LD (cm)	1.6[1.3;2.0]	1.5[1.2;1.9]	1.7[1.4;2.2]	< 0.001^*^	1.6[1.3;2.0]	1.5[1.2;1.8]	1.8[1.4;2.0]	0.004^*^	0.861
SD (cm)	1.2[1.0;1.6]	1.2[1.0;1.4]	1.2[1.1;1.7]	0.113	1.2[1.0;1.5]	1.2[1.0;1.4]	1.2[1.1;1.5]	0.137	0.376
SUV/liver	1.16[0.876;2.27]	0.968[0.769;1.1]	2.12[1.28;2.91]	< 0.001^*^	1.35[0.889;2.15]	0.913[0.781;1.17]	2.07[1.37;3.13]	< 0.001^*^	0.504
SUV/spleen	1.40[1.04;2.73]	1.12[0.9;1.29]	2.68[1.6;4]	< 0.001^*^	1.54[1.08;2.75]	1.13[0.914;1.35]	2.57[1.77;4.09]	< 0.001^*^	0.496
Gender									0.280
Female	79 (43.4%)	51(60%)	28(28.9%)	< 0.001^*^	61 (50.4%)	33(62.3%)	28(41.2%)	0.034^*^	
Male	103 (56.6%)	34(40%)	69(71.1%)		60 (49.6%)	20(37.7%)	40(58.8%)		
Lesion location									0.623
Left	125 (68.7%)	60(70.6%)	65(67%)	0.720	79 (65.3%)	38(71.7%)	41(60.3%)	0.265	
Right	57 (31.3%)	25(29.4%)	32(33%)		42 (34.7%)	15(28.3%)	27(39.7%)		
Clinical stage									0.946
I and II	47 (25.8%)	39(45.9%)	8(8.2%)	< 0.001^*^	30 (24.8%)	20(37.7%)	10(14.7%)	0.007^*^	
III and IV	135 (74.2%)	46(54.1%)	89(91.8%)		91 (75.2%)	33(62.3%)	58(85.3%)		
Primary tumor									0.352
Other cancers	71 (39%)	46(54.1%)	25(25.8%)	< 0.001^*^	40 (33.1%)	24(45.3%)	16(23.5%)	0.012^*^	
Lung cancer	111 (61%)	39(45.9%)	72(74.2%)		81 (66.9%)	29(54.7%)	52(76.5%)		

Categorical variables are presented as *n* (%), Continuous variables are presented as median (interquartile)

*PET/CT* positron emission tomography/computed tomography, *AMs* adrenal metastases, *ABNs* adrenal benign nodules, *CTU* the value on unenhanced CT, *SUVmax* the maximum standardized uptake value, *LD* long diameter, *SD* short diameter, *SUV/liver* the ratio of the adrenal, *SUVmax* to the mean liver SUV, *SUV/spleen* the ratio of the adrenal, *SUVmax* to the mean spleen SUV

^*^A significant difference between AMs and ABNs in the training or testing cohort

^+^A significant difference between the training and testing cohorts

### Comparison of PET/CT imaging features

The mean CTU of the ABN group was significantly lower than that of the AM group in both the training (25 vs. 37, *p* < 0.001) and testing (25 vs. 35, *p* < 0.001) datasets. The mean LDs of the ABN group were 1.5 cm and 1.5 cm in the training and testing datasets, respectively, which were significantly smaller than those of the AM group in both the training (1.7 cm, *p* < 0.001) and testing (1.8 cm, *p* = 0.004) datasets. The SUVmax, SUV/liver, and SUV/spleen of AMs were all significantly higher than those of ABNs in both the training and testing datasets (all *p* < 0.001) (Table 2).

### Machine-learning models

**Fig. 3:**
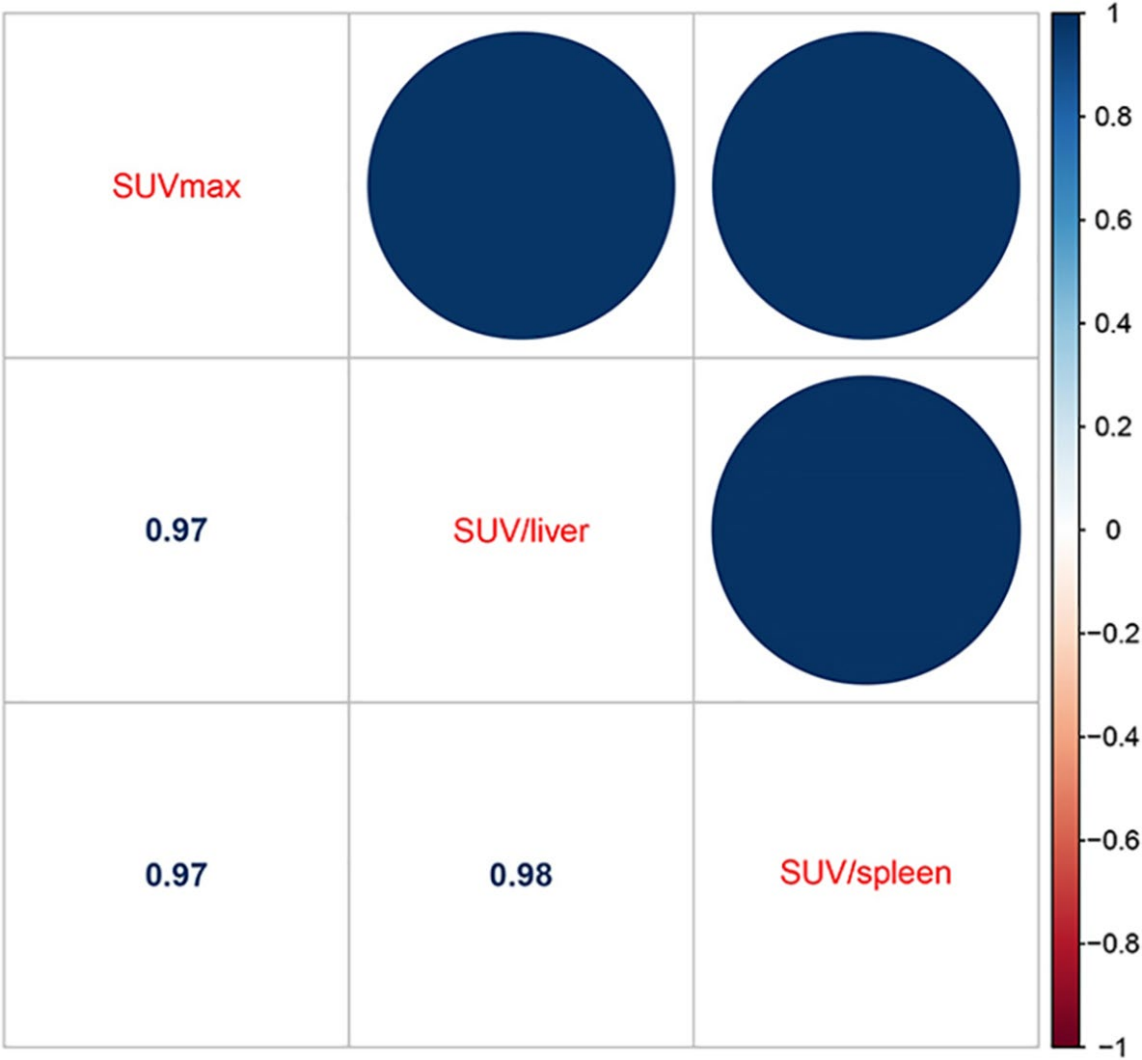
Strong correlations were observed among SUVmax, SUV/liver, and SUV/spleen

**Fig. 4:**
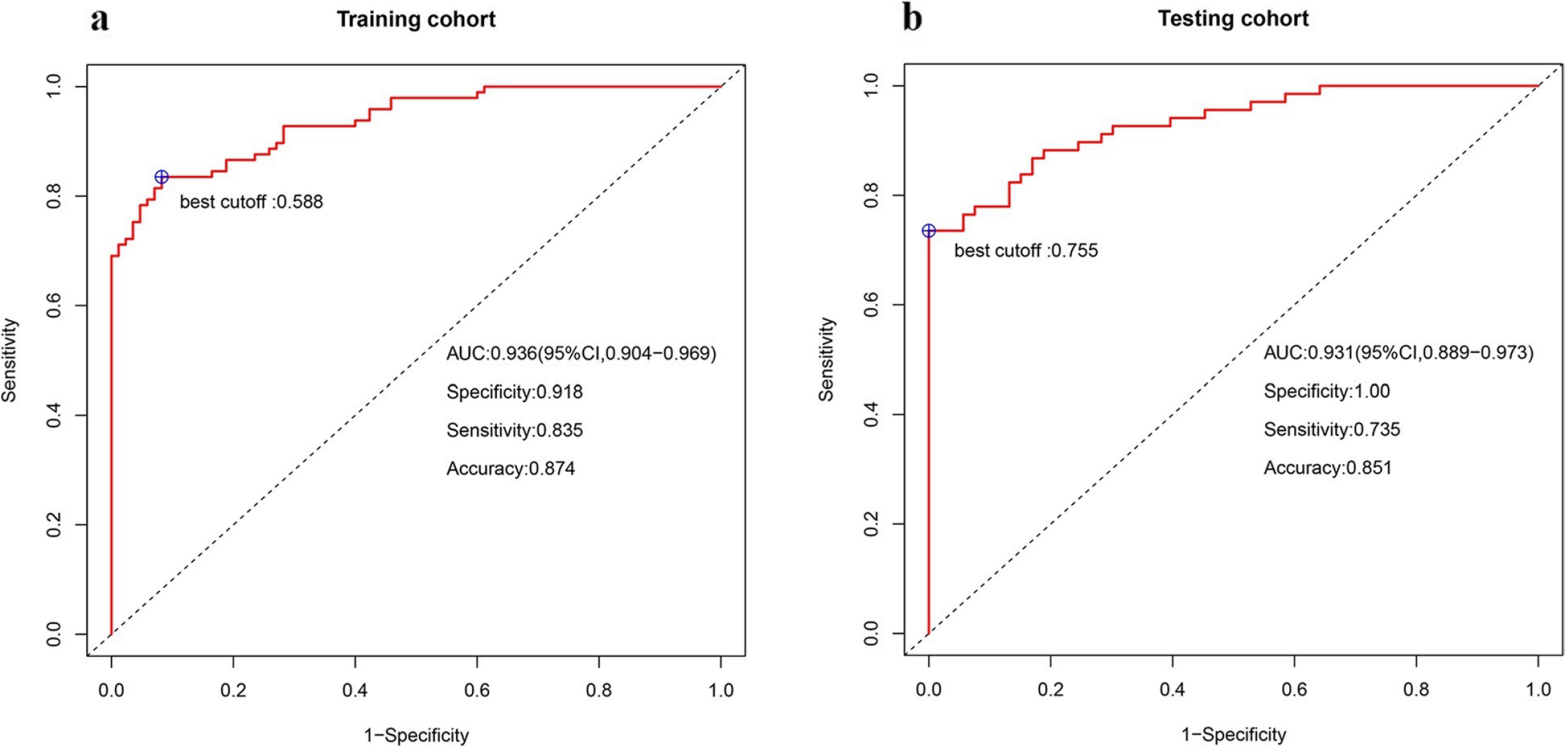
**A** The AUC of the predictive model was 0.936 [95% CI 0.904–0.969], with a sensitivity, specificity and accuracy of 0.835, 0.918, and 0.874 in the training dataset, respectively. **B** The AUC of the predictive model was 0.931 [95% CI 0.889–0.973], with a sensitivity, specificity, and accuracy of 0.735, 1.000, and 0.851 in the testing dataset, respectively

**Fig. 5:**
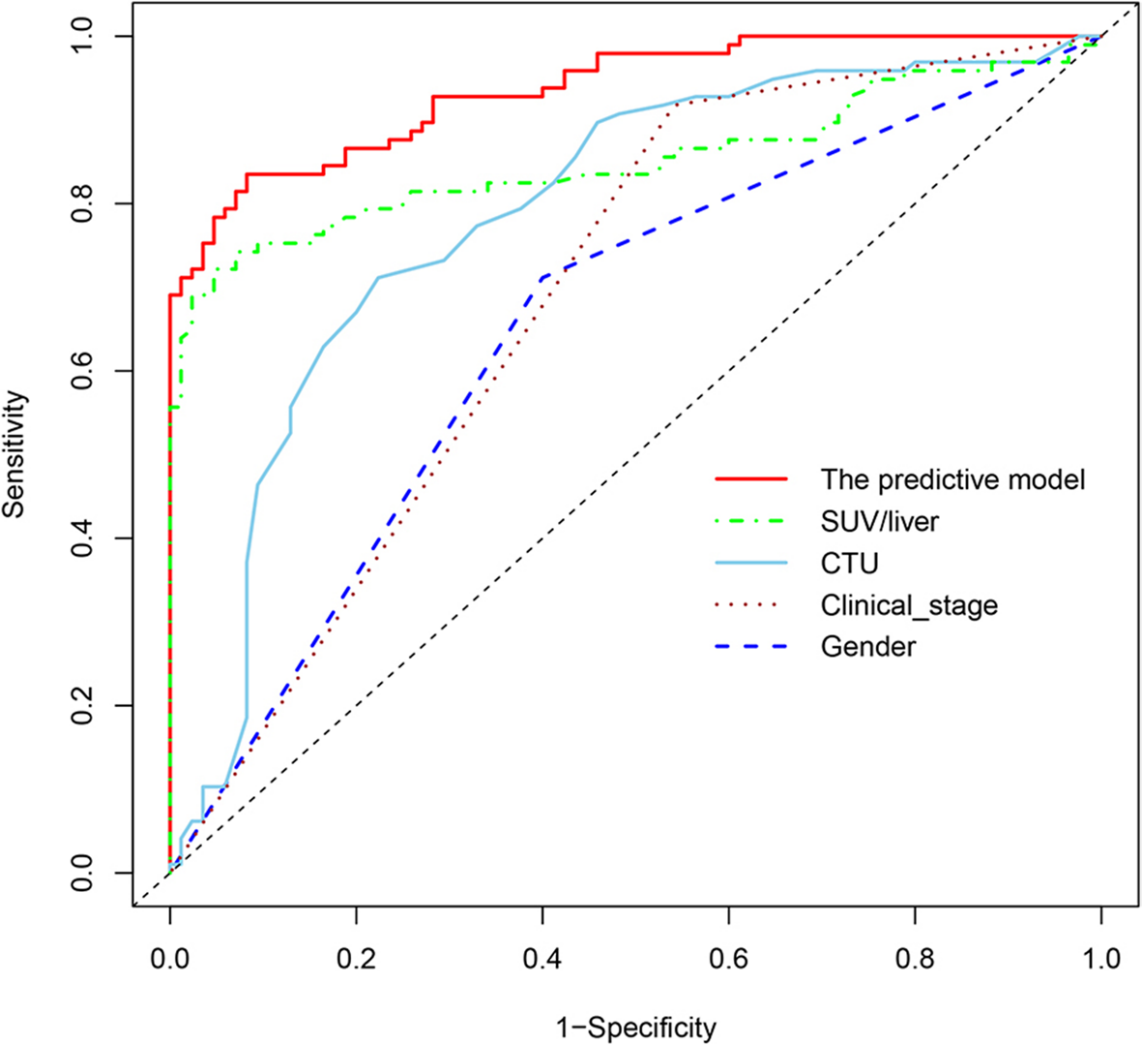
The AUC of the predictive model was higher than that of any feature alone

Since strong correlations were observed among SUVmax, SUV/liver, and SUV/spleen, only the SUV/liver feature was selected to reduce redundancies (Fig. 3). Six clinical and imaging characteristics showed significant differences between the AM and ABN groups: sex, LD, CTU, SUV/liver, and the types and clinical stage of primary cancer were used to develop a model by multivariate logistic regression. Ultimately, the predictive model revealed that sex, CTU, SUV/liver, and clinical stage of primary cancer were risk factors for AMs (Table 3). Adrenal nodules in male patients with CTU ≥ 32.5 HU, SUV/liver ≥ 1.493, and clinical stage III/IV primary cancers tended to be AMs. The predictive model achieved an AUC of 0.936 [95% CI 0.904–0.969] with a specificity, sensitivity and accuracy of 0.918, 0.835, and 0.874 in the training dataset, respectively, while yielding an AUC of 0.931 [95% CI 0.889–0.973] with a specificity, sensitivity, and accuracy of 1.000, 0.735, and 0.851 in the testing dataset, respectively (Fig. 4A, B). The AUC of the predictive model was significantly higher than that of any individual feature (all *P* < 0.05, see Fig. 5 and Table 4).

**Table 3:** Individual variables obtained from the ROC analysis for the differentiation of AMs from ABNs

Variables	Cutoff	AUC	Sensitivity	Specificity	Precision	Accuracy
Gender	–	0.656	0.659	0.711	0.600	0.670
CTU	32.5	0.788	0.742	0.711	0.776	0.784
SUV/liver	1.493	0.851	0.830	0.722	0.953	0.946
Clinical stage of primary cancer	–	0.688	0.703	0.918	0.459	0.659

*ROC* receiver operating characteristic curve, *AMs* adrenal metastases, *ABNs* adrenal benign nodules, *CTU* the value on unenhanced CT, *AUC* area under the receiver operating characteristic curve, *SUV/liver* the ratio of the adrenal, *SUVmax* to the mean liver SUV

**Table 4:** The comparison of the AUCs between the predictive model and individual risk factors

Comparison	AUC	*Z* statistic	*P*
Predictive model vs gender	0.936 vs 0.656	8.170	< 0.001
Predictive model vs CTU	0.936 vs 0.788	4.810	< 0.001
Predictive model vs SUV/liver	0.936 vs 0.851	3.754	< 0.001

*AUC* area under the receiver operating characteristic curve, *CTU* the value on unenhanced CT, *SUV/liver* the ratio of the adrenal, *SUVmax* to the mean liver SUV

**Fig. 6:**
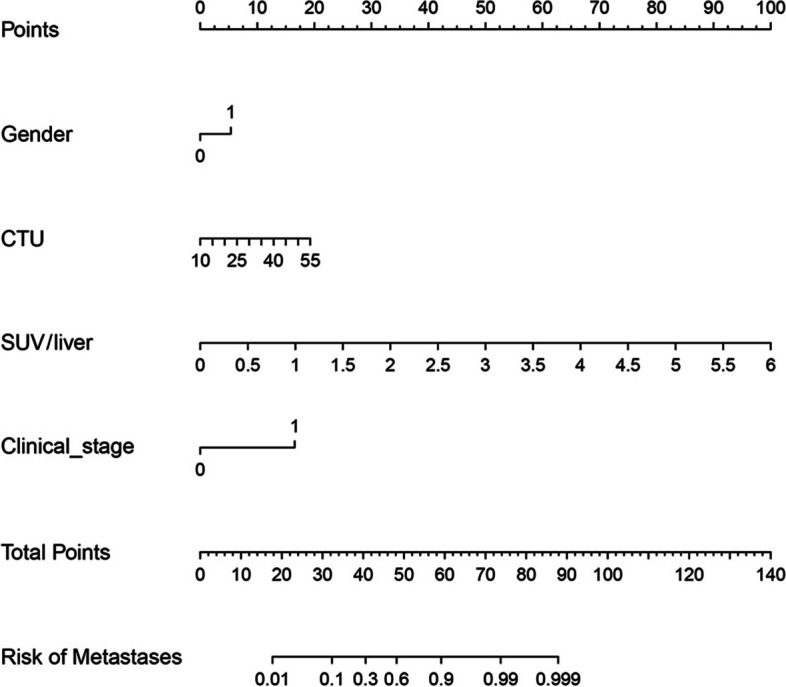
Nomogram of the predictive model

**Fig. 7:**
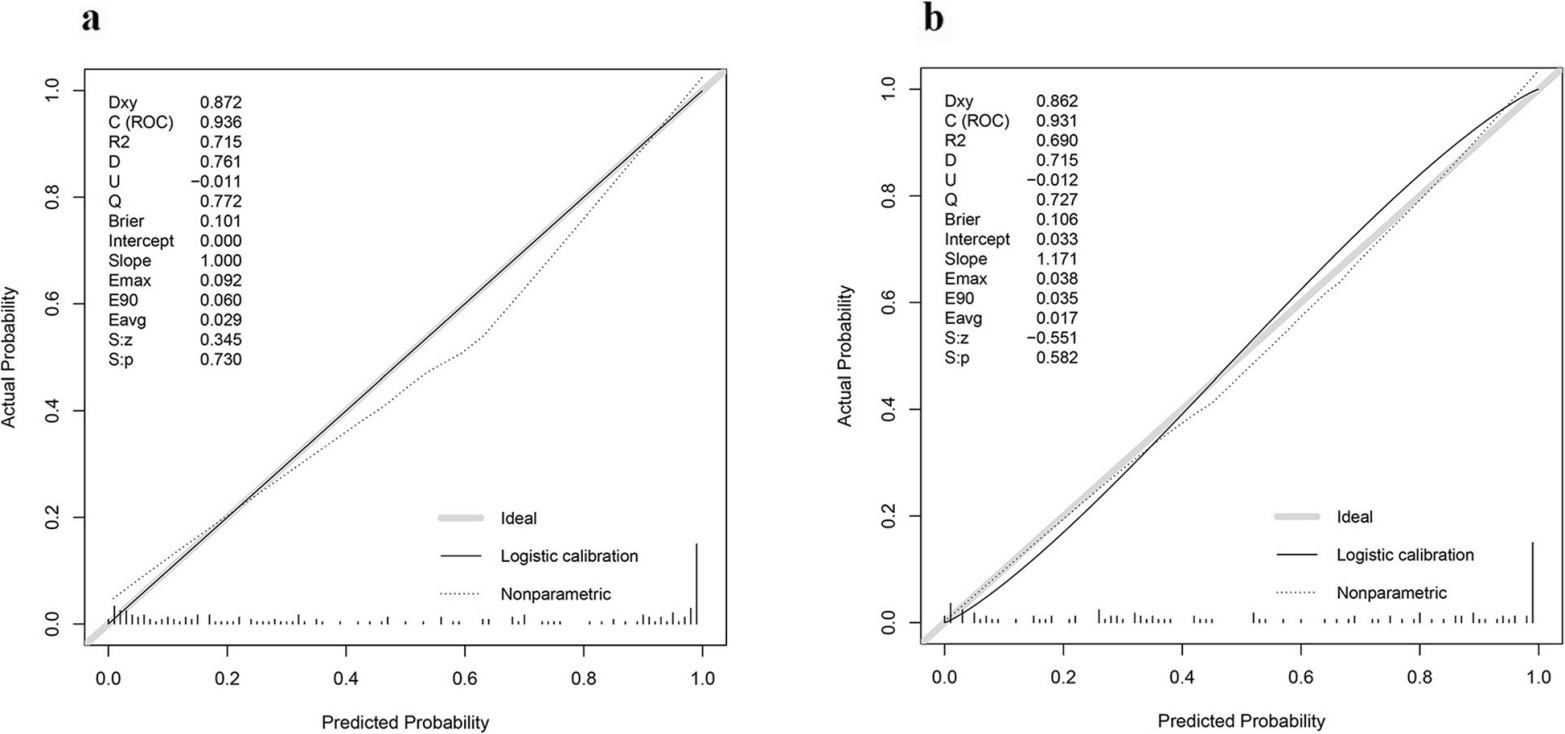
Good calibrations of the predictive model were shown in both the training (**A**) and testing datasets (**B**)

The related nomogram revealed that more than 47.615 could be considered metastases, yielding an AUC of 0.936 with a specificity, sensitivity, and accuracy of 91.8%, 83.5%, and 87.4%, respectively (Fig. 6). Good calibrations were shown in both the training and testing datasets (Fig. 7A, B).

The regression coefficients acquired from the training dataset for every individual feature in the predictive model were used to build a scoring system to facilitate the use of the model as follows:

$$\mathrm{Scoring system}=0.878\times \mathrm{Gender}+0.070\times \mathrm{CTU}+2.73\times \mathrm{SUV}\text{/liver}+2.71\times \text{Clinical staging}$$

To make it more convenient to use, we simplified the scoring system as follows:

$$\mathrm{Simplified scoring system}=1\times \mathrm{Gender}+0.1\times \mathrm{CTU}+3\times \mathrm{SUV}\text{/liver}+3\times \text{Clinical staging}$$

**Fig. 8:**
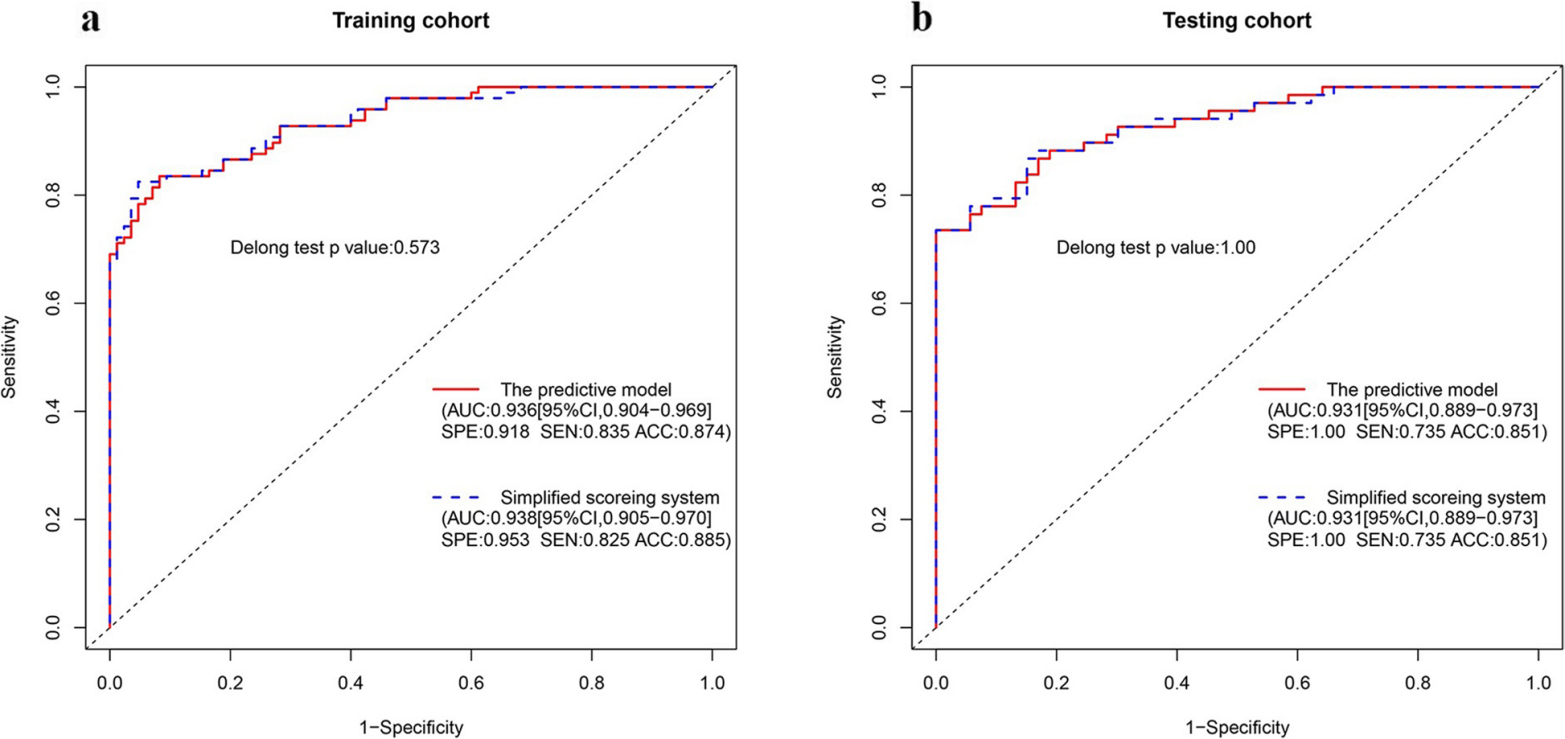
The comparison of the AUCs between the predictive model and the simplified scoring system were not significantly different in the training dataset (**A**) and testing dataset (**B**)

**Fig. 9:**
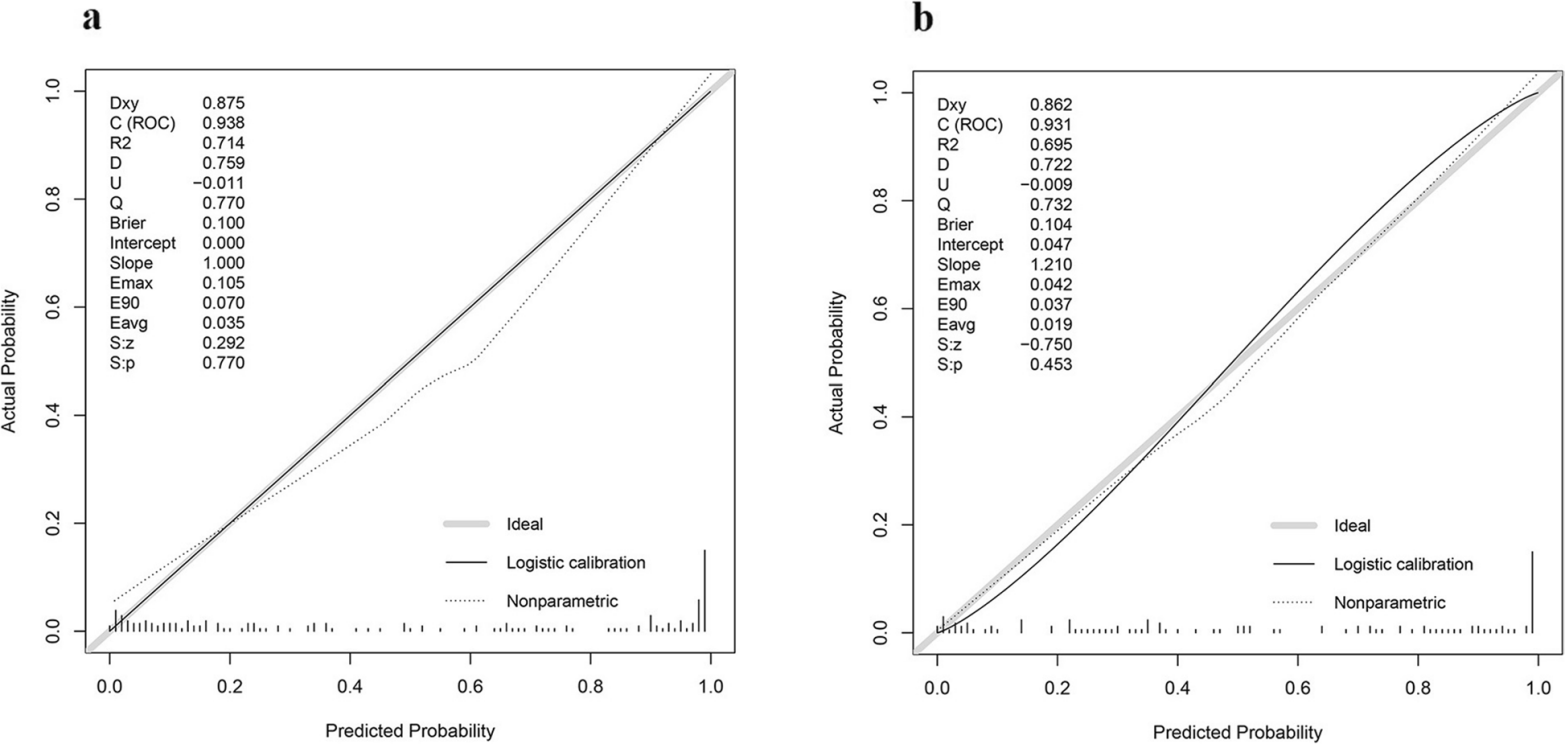
Good calibrations of the simplified scoring system were shown in both the training (**A**) and testing datasets (**B**)

The simplified regression coefficients did not affect the accuracy of differential diagnosis of the score by ROC analyses (comparations of the AUCs between the original scoring system and the simplified scoring system were not significantly different by the DeLong test in the training dataset (*P* = 0.573) and testing dataset (*P* = 1.000), Fig. 8A, B). Good calibrations of the simplified scoring system were displayed in the training dataset and testing dataset (Fig. 9A, B).

Therefore, the simplified score was obtained simply by adding 1 if the patient was male, 0 if the patient was female, 10% of the CTU and 3 times the SUV/liver and 3 if the clinical stage was III/IV. The simplified score for the patients in the training dataset ranged from 4.11 to 24.54 points. The optimal cut-off value was a score of 10.5 with a specificity and sensitivity of 95.3% and 82.5%, respectively (Table 5). This indicated that 82.5% of the AM scores were ≥ 10.5 and that 17.5% of AMs would be missed at this cut-off value. Lower cut-off values may be used to reduce the proportion of missed diagnoses. The rate of missed diagnosis could be as low as 2.1% at the cost of a low precision (69.3%) when the cut-off was set at 7.5. Overall, the higher the score obtained by this simplified scoring system, the greater the risk of the lesion being predicted as metastasis. The simplified scores for patients in the testing dataset were shown in Table 6.

**Table 5:** Cut-off values and corresponding performance data for the simplified scoring system in the training dataset

Cutoff	Sensitivity (95% CI)	Specificity (95% CI)	Precision (95% CI)	Accuracy (95% CI)
6	1.00(1.00,1.00)	0.235(0.145,0.325)	0.599(0.523,0.674)	0.643(0.640,0.645)
6.5	1.00(1.00,1.00)	0.294(0.197,0.391)	0.618(0.542,0.694)	0.670(0.668,0.673)
7	0.979(0.951,1.00)	0.388(0.285,0.492)	0.646(0.569,0.724)	0.703(0.701,0.706)
7.5	0.979(0.951,1.00)	0.506(0.400,0.612)	0.693(0.616,0.771)	0.758(0.756,0.760)
8	0.928(0.876,0.979)	0.600(0.496,0.704)	0.726(0.647,0.804)	0.775(0.773,0.777)
8.5	0.918(0.863,0.972)	0.718(0.622,0.813)	0.788(0.712,0.863)	0.824(0.823,0.826)
9	0.866(0.798,0.934)	0.765(0.675,0.855)	0.808(0.732,0.883)	0.819(0.817,0.820)
9.5	0.845(0.773,0.917)	0.824(0.742,0.905)	0.845(0.773,0.917)	0.835(0.834,0.837)
10	0.835(0.761,0.909)	0.894(0.829,0.960)	0.900(0.838,0962)	0.863(0.861,0.864)
10.5	0.825(0.749,0.900)	0.953(0.908,0.998)	0.952(0.907,0.998)	0.885(0.884,0.886)
11	0.742(0.655,0.829)	0.976(0.944,1.00)	0.973(0.936,1.00)	0.852(0.850,0.853)
11.5	0.711(0.621,0.802)	0.988(0.965,1.00)	0.986(0.958,1.00)	0.841(0.839,0.842)
12	0.680(0.588,0.773)	1.00(1.00,1.00)	1.00(1.00,1.00)	0.830(0.828,0.831)
12.5	0.598(0.500,0.696)	1.00(1.00,1.00)	1.00(1.00,1.00)	0.786(0.784,0.788)
13	0.536(0.437,0.635)	1.00(1.00,1.00)	1.00(1.00,1.00)	0.753(0.751,0.755)

*CI* confidence interval

**Table 6:** Cut-off values and corresponding performance data for the simplified scoring system in the testing dataset

Cut-off	Sensitivity (95% CI)	Specificity (95% CI)	Precision (95% CI)	Accuracy (95% CI)
6	1.00(1.00,1.00)	0.170(0.069,0.271)	0.607(0.517,0.698)	0.636(0.633,0.640)
6.5	1.00(1.00,1.00)	0.302(0.178,0.425)	0.648(0.556,0.739)	0.694(0.691,0.698)
7	1.00(1.00,1.00)	0.340(0.212,0.467)	0.660(0.569,0.752)	0.711(0.707,0.714)
7.5	0.985(0.957,1.00)	0.377(0.247,0.508)	0.670(0.578,0.762)	0.719(0.716,0.722)
8	0.971(0.930,1.00)	0.434(0.301,0.567)	0.688(0.595,0.780)	0.736(0.732,0.739)
8.5	0.941(0.885,0.997)	0.547(0.413,0.681)	0.727(0.634,0.820)	0.769(0.766,0.771)
9	0.912(0.844,0.979)	0.698(0.575,0.822)	0.795(0.705,0.884)	0.818(0.816,0.821)
9.5	0.868(0.787,0.948)	0.830(0.729,0.931)	0.868(0.787,0.948)	0.851(0.849,0.853)
10	0.794(0.698,0.890)	0.887(0.801,0.972)	0.900(0.824,0.976)	0.835(0.832,0.837)
10.5	0.779(0.681,0.878)	0.943(0.881,1.00)	0.946(0.887,1.00)	0.851(0.849,0.853)
11	0.735(0.630,0.840)	1.00(1.00,1.00)	1.00(1.00,1.00)	0.851(0.849,0.853)
11.5	0.662(0.549,0.774)	1.00(1.00,1.00)	1.00(1.00,1.00)	0.810(0.807,0.812)
12	0.574(0.456,0.691)	1.00(1.00,1.00)	1.00(1.00,1.00)	0.760(0.757,0.763)
12.5	0.500(0.381,0.619)	1.00(1.00,1.00)	1.00(1.00,1.00)	0.719(0.716,0.722)
13	0.456(0.338,0.574)	1.00(1.00,1.00)	1.00(1.00,1.00)	0.694(0.691,0.698)

*CI* confidence interval

Next, patients with histologic confirmation (AMs 15, ABNs 59), as a new dataset, were further analyzed. The simplified scores for the patients with histologic confirmation ranged from 3.63 to 21.53 points. When using the optimal cut-off value of 10.5, the specificity and sensitivity of the simplified scoring model were 96.6% and 86.7%, respectively, and accuracy was up to 94.6%, which indicates that compared with the original training and testing datasets, the simplified scoring system still has good diagnostic performance in the confirmed dataset (Table 7).

**Table 7:** Cut-off values and corresponding performance data for the simplified scoring system in the confirmed dataset

Cut-off	Sensitivity (95% CI)	Specificity (95% CI)	Precision (95% CI)	Accuracy (95% CI)
6	1.00(1.00,1.00)	0.220(0.115,0.326)	0.246(0.138,0.354)	0.378(0.372,0.385)
6.5	1.00(1.00,1.00)	0.305(0.188,0.423)	0.268(0.152,0.384)	0.446(0.439,0.452)
7	1.00(1.00,1.00)	0.390(0.265,0.514)	0.294(0.169,0.419)	0.514(0.507,0.520)
7.5	1.00(1.00,1.00)	0.475(0.347,0.602)	0.326(0.191,0.462)	0.581(0.575,0.588)
8	1.00(1.00,1.00)	0.508(0.381,0.636)	0.341(0.201,0.481)	0.608(0.602,0.614)
8.5	1.00(1.00,1.00)	0.627(0.504,0.751)	0.405(0.247,0.564)	0.703(0.697,0.708)
9	0.933(0.807,1.00)	0.729(0.615,0.842)	0.467(0.288,0.645)	0.770(0.766,0.775)
9.5	0.933(0.807,1.00)	0.831(0.735,0.926)	0.583(0.386,0.781)	0.851(0.848,0.855)
10	0.867(0.695,1.00)	0.898(0.821,0.975)	0.684(0.475,0.893)	0.892(0.889,0.894)
10.5	0.867(0.695,1.00)	0.966(0.920,1.00)	0.867(0.695,1.00)	0.946(0.945,0.947)
11	0.800(0.598,1.00)	0.983(0.950,1.00)	0.923(0.778,1.00)	0.946(0.945,0.947)
11.5	0.733(0.510,0.957)	0.983(0.950,1.00)	0.917(0.760,1.00)	0.932(0.931,0.934)
12	0.667(0.428,0.905)	1.00(1.00,1.00)	1.00(1.00,1.00)	0.932(0.931,0.934)
12.5	0.600(0.352,0.848)	1.00(1.00,1.00)	1.00(1.00,1.00)	0.919(0.917,0.921)
13	0.533(0.281,0.786)	1.00(1.00,1.00)	1.00(1.00,1.00)	0.905(0.903,0.908)

*CI* confidence interval

## Discussion

For patients with extra-adrenal malignancies, AMs and ABNs are both common tumors. If adrenal lesions are found during cancer follow‐up or staging in cancer patients, an accurate diagnosis is extremely important for planning the treatment. Recently, a few studies on adrenal masses have shown the potential of textural features and radiomics for differentiating AMs from ABNs [17, 18]. However, radiomics has not been extensively applied in clinical practice owing to time-consuming computation and analysis for high-dimensional characteristics. Therefore, the work-up of adrenal lesions, especially indeterminate adrenal nodules, mainly depends on the traditional imaging parameters estimated by the human eye [24–27]. In this study, our predictive model developed with the clinical manifestations (clinical stage of primary malignancies and sex) and traditional PET/CT imaging features (CTU and SUV/liver of adrenal nodules) had robust performance in effectively distinguishing the two groups. Meanwhile, the simplified scoring system had comparable diagnostic value to the predictive model (or nomogram) and great foregrounds in clinical practice due to its simplicity and convenience.

Sex and clinical stage of primary malignancies were independent clinical factors for differentiating AMs from ABNs in our study. AMs were more likely to occur in men in our study, which was consistent with the results of Chen et al. [28], who found that the proportion of female patients with AMs was significantly lower than that with ABNs. There may be two primary reasons for the difference in the sex ratio between AMs and ABNs. First, lung cancer, accounting for the highest ratio of AMs, is most likely to occur in men [29]. Second, the incidence of gynecological and breast and thyroid cancers accounting for the highest ratio of ABNs was notably high among women [30, 31]. Regarding the clinical stage of primary cancers, ABNs were more likely to be in the early stage (I/II), while AMs were more likely to be in the late period (III/IV), which was in accordance with previous reports [32, 33].

In our study, the SUVmax, SUV/liver, and SUV/spleen were all higher for AMs than for ABNs in both the training and testing datasets (*P* were all < 0.001). Since strong correlations were observed among SUVmax, SUV/liver, and SUV/spleen, we chose SUV/liver as a risk factor for reducing redundancy according to a previous study [34]. The AUC, sensitivity, and specificity of SUV/liver in the differentiation of AMs from ABNs in this study were 0.851, 83.0%, and 72.2%, respectively, which were all lower than those in previous studies. Watanabe et al. found that the AUC of SUV/liver was 0.99 with a sensitivity of 96% and a specificity of 100% in the differential diagnosis of adrenal metastases and adrenal adenomas [34]. Boland et al. reported that SUV/liver had 100% specificity and sensitivity for differentiating adrenal lesions as malignant or benign in cancer patients based on PET images [35]. In addition, the best cut-off value of SUV/liver was 1.493 in this study, which was higher than that of Watanabe et al. (1.37) [34] and Launay et al. (1.33) [20]. The reason for these may be due to our special research object: indeterminate adrenal nodules, and differential diagnosis of indeterminate adrenal nodules has always been a difficult point in daily imaging and clinical practice. At present, there are few studies based on PET/CT about this special research object. In our study, the CTU of AMs was significantly higher than that of ABNs based on PET/CT, which was consistent with a previous study [20, 21]. Kunikowska et al. [21] showed that malignant adrenal tumours had a significantly higher mean CT attenuation value than benign tumours on PET/CT. Moreover, a CTU > 32.5 HU was an independent predictor of AMs with a sensitivity of 78.8% and a specificity of 74.2% in our study.

Most previous reports only focused on individual PET/CT imaging features to distinguish AMs and ABNs [20–22]. Our study was unique because of the comprehensive analysis of traditional clinical and PET/CT imaging features focused on indeterminate adrenal nodules. We found that the AUC for the predictive model combining the clinical stage of extra-adrenal cancers, sex, CTU, and SUV/liver of adrenal nodules reached 0.936, with a specificity, sensitivity, and accuracy of 91.8%, 83.5%, and 87.4% in the training dataset, respectively, while yielding an AUC of 0.931 and a sensitivity, specificity, and accuracy of 73.5%, 100%, and 85.1% in the testing dataset, respectively. Although these traditional clinical and PET/CT imaging features have relatively lower diagnostic accuracy and are not specific for metastases when used alone, the ability of differential diagnosis was significantly improved when these characteristics were combined in our study. Meanwhile, the simplified scoring system showed a good ability to differentiate AMs from ABNs with AUCs of 0.938 and 0.931, respectively, in the training and testing datasets, which had comparable diagnostic value to the predictive model (or nomogram), and the comparison of AUCs between the predictive model (or nomogram) and the simplified scoring system was not significantly different in either the training or testing datasets. When using the nomogram to predict the risk of metastases, we needed to acquire the points of each feature using a naked eye comparison, which would easily lead to an inaccurate total score and an inaccurate ultimate predictive percentage. We could directly gain the score of every feature without visual comparison in the simplified scoring system. Hence, the simplified scoring system has great foregrounds in clinical practice due to its simplicity and convenience.

We believe this may be a valuable predictive model for diagnosing AMs. Therefore, biopsy could be avoided in some cases.

But there is a crucial point to note when using this model. In clinical practice, our proposed model may provide management recommendations for patients with indeterminate adrenal nodules and extra-adrenal malignancies. Generally, a higher score obtained from this simplified scoring system suggests an increased likelihood of the lesion being predicted as a metastasis. However, increasing the cut-off value reduces sensitivity, potentially leading to fail to detect metastases. This may result in inaccurate clinical staging of the primary cancer and impact the formulation of precise treatment plans. On the other hand, lowering the cut-off point reduces the rate of missed diagnoses but lowers specificity, which increases the risk of falsely detecting ABN as AM. This could result in unnecessary examinations and psychological pressure for patients. Therefore, we recommend engaging in careful communication with patients before establishing treatment protocols, as the choice of the cut-off value should depend on the level of risk both patients and doctors are willing to accept. Ultimately, the simplified scoring system serves as a convenient and valuable tool, providing evidence for doctors and patients.

There were several limitations. Firstly, features such as CTU and SUVmax may be different among radiologists since they were manually extracted. Experienced radiologists may have more accurate measurement results than junior radiologists. Measuring such features through computer algorithms may improve the stability and reproducibility of models and features. Secondly, since only a small number of patients underwent pathological confirmation, the majority of adrenal nodules in our study were diagnosed based on follow-up imaging. However, this situation reflects current practices. Meanwhile, based on the confirmed dataset, we further validated the good diagnostic performance of our predictive model. Thirdly, in our study, thick-slice PET and CT images were utilized, and contrast-enhanced CT was not included as part of the PET/CT scanning procedure. It is important to note that previous studies have demonstrated a notable improvement in diagnostic accuracy when PET data is combined with contrast-enhanced CT data [36]. Therefore, conducting a subsequent study incorporating thin-slice enhanced CT scans may potentially further enhance the overall performance of this model. Fourthly, this study was retrospective and single-center with a relatively small sample size, which only included the Asian population, and the coefficients may only apply to one PET/CT system with one acquisition method and one reconstruction method. Further multi-center studies with multiple races and large sample sizes and different PET/CT systems may improve the performance of this predictive model to a certain extent.

## Conclusions

In summary, the predictive model and simplified scoring system based on traditional clinical and PET/CT imaging features showed good diagnostic performance for differentiating AMs from ABNs in cancer patients with indeterminate adrenal nodules and thus assisted clinicians in pre-treatment decision-making.

## Data Availability

The data sets analyzed during the current study are not publicly available for patient privacy purposes but are available from the corresponding author on reasonable request.

## Abbreviations

PET/CT: Positron emission tomography/computed tomography
AMs: Adrenal metastases
ABNs: Adrenal benign nodules
SUVmax: The maximum standardized uptake value
SUV: Standardized uptake value
AUC: Area under the receiver operating characteristic curve
LD: Long diameter
SD: Short diameter
CI: Confidence interval
ROI: Region of interest
HU: Hounsfield units
CTU: The value on unenhanced CT
ROC: Receiver operating characteristic curve
MRI: Magnetic resonance imaging
SUV/liver: The ratio of the adrenal SUVmax to the mean liver SUV
SUV/spleen: The ratio of the adrenal SUVmax to the mean spleen SUV
